# Report on the Trial of Jonathan Martin, for Setting Fire to the York Minster

**Published:** 1830

**Authors:** 


*Report of the Trial of Jonathan Martin, for setting fire to the York Minister.* The destruction of the venerable cathedral of York, and the protracted trial of the incendiary, produced very considerable excitement at the time in England. Not a doubt existed as to the fact, but his examinations produced an universal conviction on the minds of medical men, that he was a decided monomaniac. We copy from the Medico-Chirurgical Review, the report of the testimony of two of the witnessses examined on the trial.

*Caleb Williams, on his affirmation—examined by Mr. Sergeant Jones.*—Q. You are a surgeon in the city of York, I believe ? A Yes. Q. Are you one of the medical attendants at the lunatic asylum, called the Friend's Retreat ? A. Yes. Q. Have you been conversant on the subject of the diseases of lunatics ? A. Yes, for five or six years. Q. When did you first of all see the prisoner at the bar, Martin, so as to observe his state of mind ? A. On the 21st of the present month. Q. How often have you seen him since that time ? A. Daily, with the exception of two days. Q. In the first place I would ask you, what appears to be the state of his mind ? A. I consider him to be what is denominated a "*monomaniac.*" *Baron Hullock.* Just state, for the information of the jury, what that is. A. It is a species of insanity, in which the delusion is confined to one idea only, or to one train of ideas. Q. Is that the expression that is well known and commonly used in medical treatises and discussions ? A. Yes. Q. It is opposed to delirium or delusionn, upon all subjects generally ? A. Yes. Q. Then first of all I ask you, whether you have taken pains to satisfy yourself, whether the symptoms or appearances of his insanity are feigned or bona fide ? A. I believe they are bona fide really. Q. Have you had any discourse with him on the subject of dreams ? A. I have. Q. What is your impression with regard to the influence of dreams upon the mind of the prisoner ? A. I believe his dreams to have more influence over him than they would have over a person of sound mind. Q. To what subject do the dreams of the prisoner apply ? A. Chiefly to the shbject of religion. Q. Did you also attend to his bodily strength ? A. I did. Q. Are there certain symptoms connected with his bodily strengththat afford any influence with regard to the state ot his mind ? A. There are. Q. Did the appearances which you witnessed with regard to the state of his body, concur, or run differently, with his state of mind ? what is your opinion with regard to the state of his mind, as derived from those appearances ? A They confirmed my opinion with regard to the state of his mind. Q. How was the eye ? A. The eye was red. Q. I need hardly ask you, whether persons acquainted with medical subjects of this sort, look to the eye of the patient as a test of their state of mind ? A. The eye is frequently observed, certainly, and the countenance generally. Q. How was he as to his pulse ? A. It was full, hard, strong, and quicker than natural. Q. Is that state of the pulse the one commonly found in instances of monomania ? A. Yes, during the period of excitement it is common. Q. Do you think him a person that it would be safe to trust to go abroad ? A. I think the instances that have been given, prove that it is not safe for him to go abroad. Q. Then you are of opinion that he is insane ? A. Yes. Q. And that his madness is upon one particular subject, or upon one train of ideas ? A. Yes. Q. Are there many patients or persons in confinement who are laboring under monomania, and who have no other complaint? A. Yes, there are many ; I have opportunities of seeing many every week. *Baron Hullock.* Were it otherwise, he would not be denominated a monomaniac.

*Crossexamined by Mr. Alderson.* Q. Are not these monomaniacs capable of distinguishing right from wrong ? A. Not upon subjects upon which they are deluded. Q. But upon general subjects ? A. Upon subjects perfectly unconnected with their delusion they have the power of judging of right from wrong. Q. Upon those subjects upon which they are capable of so judging, they act like other persons capable of judging right from wrong ? A. Yes, frequently. Q. Do they avoid dangers consequent upon their actions ? A. Frequently. Q. Do they run away after having done a thing that they know is wrong ? A. Very commonly. Q. Do they adopt skilful means to avoid the consequence of such an act ? A. They are very cunning in escaping punishment. Q. And also very cunning in pursuing the means to accomplish an act ? A. Yes, the same. Q. You don't consider that that shows that they know, beforehand, that they are doing a wrong act ? A. No. Q. In what way would they show they were doing a wrong act : would they do it by staying, if they did know they had done a wrong act ? In what way do you, as a medical man, expect that insane persons would show it ? A. I should think that an insane man would endeavor to avoid being detected. Q. Then it is possible for a person, being an insane man, to do an act which he knows will be followed by punishment, and consequently endeavor to avoid it ? A. It is Q. Does not the fear of punishment deter them ? A. I believe the mind is so absorbed with the delusive idea, that no other idea occupies their attention. Q. Do you not know that persons who are insane are prevented from doing such acts through fear of punishment ? A. No, that will not operate so as to prevent the delusion coming on. Q. What, in your judgment, is the peculiar subject upon which this person, the prisoner, is to be looked on as being mad ? A. An undue reliance upon his dreams, believing they are revelations of divine will, directing him to perform certain acts. Q. Has he told you that that is so ? A. Yes. Q. Have you any other reason for knowing it, than that he has told you so ? A. No. Q. Had you any other conversations with him before ? A. No. Q. When did you first see him ? A. On the 21st of this month. Q. That was the first day of the assizes ? A. Yes. Q. All your interviews have been with him since the judges came to town ? A. Yes. Q. Since that he has been telling you of his dreams. A. Yes.

*Reexamined by Mr. Brougham.* Q. You say you have had considerable experience in regard to the diseases of insane persons, from your attending a lunatic asylum ? A. Yes. Q. Do you consider, judging from your experience, that the conduct and appearance of this prisoner could have been put on with the view of deceiving you, or that it was otherwise than real ? A. I don't think it could.

*Dr. Baldwyn Wake sworn—examined by Mr. Brougham.* Q. You are a physician in York. A. Yes. Q. Have you had experience of lunatic patients ? A. Yes. Q Are you physician to a lunatic establishment ? A. Yes, I am ; to the York Lunatic Asylum. Q. How long have you been so ? A. For thirteen years and upwards. Q. Have you seen the prisoner lately ? A. Since the 21st of this month, and also about three or four weeks ago, without his being aware I did see him. I looked through the window of Mr. Kilby's apartment. Q. Have you paid attention to his case lately ? A. Yes, I devoted an hour or two at a time, and investigated his case as carefully as time would allow me. Q. During the visits you have made, and you have seen him almost daily, you have investigated his case ? A. Almost daily ; and I have seen him occasionally twice a day. Q. Now, judging from what you have seen of him, and applying the result of your own experience in such diseases to him, is it your opinion that he is of sound or unsound mind ? A. Of unsound mind, most undoubtedly. Q. Of what description is the insanity he labors under ? A. *Monomania.* Q. Is that a frequent species of the complaint ? A. Very frequent ; there are a great many varieties, Q. But the characteristic of the whole of them is, that of being under a delusion on one particular point, and more or less sane upon other points ? A. Yes, sir ; that they have sanity upon most other points. Q. Did you observe any particular bodily infirmity about him ? A. Yes. Q. Had you occasion to observe his head ? A. I had. Q. What did you observe in regard to it ? A. I observed that it has the appearance, on the frontal bone, as if he had had a wound or accident, and upon enquiring as to it, he told me a long detail of his burning the Minster, nearly as has been stated before ; and in doing so, I observed that he suddenly put his hand to his head, and seemed quite lost for some time ; that attracted my attention more particularly towards his head, and when he was apparently sufficiently recovered to an swer questions, I asked him what was the matter, and he said, "when I talk much, a pain strikes me at that place where I had received a wound." Q. Did you, in consequence of that observation, examine his head more particularly? A. Yes, I did. Q. What was the result of your examination? A. I found there was the mark of an injury there, and I enquired into the particulars of it. Q. I shall not trouble you to give a minute description of it, but you say there was apparently the remains of an injury? A. Yes. Q. Did you feel the marks of that injury with your hand? A. I did. Q. What effect did the pressure of your hand make upon it? A. Not mnch effect; but I found that there seemed to be some little depression upon that part, and I enquired something further about it. Q. I shall not ask you what he told you; did you make any enquiry as to his appetite? A. I did, and I found thnt he had a most voracious appetite. I went down in the cell when his wife came to him with his dinner, and I observed that he eat in the most voracious manner a beef stake pie, and he devoured it in a short time, although it appeared as much as any three men could have eaten. Q. Is that a symptom frequently attending the species of insanity you describe? A. It is an attendant symptom upon insanity generally, where the health is at the same time good. There is generally a voracious appetite, and he devours his food in a particular manner. Q. Did you observe his eye? A. I did. Q. What appearance had that? A. It was glassy, and when he was excited, it betrayed a great deal of *maniacal* expression, or appearance in it, which would strike any person accustomed to those diseased patients, so that they cannot mistake the nature of their complaint. Q. Could those bodily symptoms that you describe, have been put on for the purpose of deceiving you? A. I presume not. Q. Don't you believe they could not? A. I do firmly believe they could not; it is a mistake when I use the other expression, that I presumed it could not be assumed. Q. Had the pulse any thing to do with discovering the state of the patient? A. The pulse was uncommonly hard, and not very frequent. I examined the state of the heart, but I found the action of the heart did not account for the strong pulse, I therefore attributed it to his delusion, and to the state of his mind. That symptom is frequently attendant upon insanity, when there is a restlessness and excitement. Q. Is an inability to remain at rest one of the symptoms? A. I observed him when he did not know it, and perceived him walk up and down apparently five or six miles an hour; and that was his usual manner. Q. Was that observed frequently? A. Yes. Q. From the opportunities you had of seeing him upon those occasions you speak of, have you any doubt that he is a person of unsound mind? A. I have no doubt of it. Q. Is he a person capable of distinguishing right from wrong? A. Upon the subject of his delusion, I think not. I could state my reason for drawing that conclusion, if necessary, from another case that bears upon this one. Q. No, I don't ask you that, but I may ask you, if a person, who has a delusion upon one particular subject, and is, upon that subject, incapable of distinguishing right from wrong, be capable, upon other subjects, of distinguishing right from wrong A. Generally, they can do so upon other subjects. Q. Could you, upon other subjects, rely, even with certainty, on their distinguishing right from wrong? A. Yes. Q. Upon subjects generally, excluding those to which this delusion applies, I ask you, are insane persons under the influence of fear? A. I think upon maniacs in general, fear operates very powerfully. Q. And will they endeavor to effect their escape after they have done a wrong act? A. Yes, they will endeavor to elude punishment. Q. May a person laboring under this species of disease, called "monomania," be incapable of distinguishing right from wrong, and then doing that which we should call wrong, be able afterwards to know that they are likely to be punished for it? A. I think that when that delusion is frequently excited, a maniac does not know right from wrong; but then, after he has committed a crime, he may become sensible of his danger. I have known many instances of monomania, and I know that at certain times they have lucid intervals, and will be conscious of the error they have committed, and the delusion under which they labour; of this I know a striking case, if it were necessary upon this occasion to state it.

Baron Hullock summed up in a long, elaborate, and able manner. The fact of Martin's having set fire to the Minster was clear as the sun at noonday. But the question of culpability—that is to say, of sanity or insanity, rested entirely, or almost entirely, on the medical witnesses. The jury instantly acquitted him, on the plea of insanity—and every man of the slightest medical infermation, must acquiesce in the decision. We tender our thanks and the profession of sincere esteem to Dr. Baldwyn Wake and Mr. Williams.

We have adverted to this case not so much with the view of enlightening the mind of the profession on the subject of monomania, for the subject of Insanity generally occupies so important a place among the elementary studies of medicine, that few physicians of any science are unacquainted with the general principles of the subject, at least as a matter of scientific speculation; but for the purpose of urging them to embrace every opportunity of acquiring practical information upon this complicated and intricate question, and of impressing upon them the importance of disseminating correct information upon this subject.

Few persons are unaware of the fact that mental alienation may be infinitely diversified both in its symptoms and in its seat—that every faculty of the mind is liable to be perverted or prostrated, exhibiting every conceivable variety of partial insanity.

So insensible are the gradations of mental aberration that it is frequently very difficult to draw the distinction between partial insanity, and peculiarity of temperament or manners; and when the former has been established, men of the greatest sagacity, and the most profound investigation, have failed to say where moral responsibility should cease.

The subject is thus frequently surrounded with difficulties, when divested of every circumstance calculated to elicit feeling, or enlist prejudice. When questions of this nature, therefore are brought before judicial tribunals, where very powerful prejudices, passions and interests frequently combine their influence to mislead public opinion, it would be indeed a matter of surprise, if its decisions were not frequently very unjust and oppressive. The lives and property of individuals, the peace and happiness of families, frequently depends upon this decision. Since therefore, correct and definite information upon this subject is confined almost entirely to those who study it professionally, it becomes the peculiar duty of physicians to correct the erroneous impressions which are so current in society.

The varieties and degrees of insanity are, as we before remarked, innumerable. In some rare instances, the different faculties of the mind, perception, imagination, judgment, and the moral and active faculties, are almost equally affected, and sometimes are so completely prostrated, that the individual is destitute of those feeble glimmerings of reason, and even of those instincts which attend the lower forms of animal life. Far more frequently, however, reason is more partially affected. Reason fancies herself at the helm, and still appears to maintain her supremacy, but she is hurried away by passion, misled by prejudice, spell bound by some prevailing hallucination, or distracted by false perceptions. "Distraction" says Erskine "sits beside her, holds her trembling on her seat, and frightens her from her propriety." Her efforts are still exerted, but it is under the direction, and in the defence of her enemies. Such a man often reasons logically, and his deductions are false, only because his premises are the creatures of his imagination.

In partial insanity, the derangement is frequently confined to one particular subject, or train of ideas, which forms the subject of their delusion. Not only do such persons converse rationally upon ordinary subjects, but even on their peculiar hallucination they manifest great ingenuity and sagacity in defence of their false positions. The mind constantly directed to one subject, and stimulated by a morbid excitement, acquires a collection of facts, and a sagacity of applying them, far superior to what they possessed in health, and which is perfectly astonishing to those who are not familiar with the diseases of the intellect; and when subjected to a judicial investigation, they frequently avoid with the greatest art and address, the subject of their delusion. The close connection between madness and a certain species of wit, is one of those poetic truths which is confirmed by every day observation ; and cunning, so far from being an evidence of a sane mind, is frequently a characteristic of insanity.

Those who are accustomed to reflect upon the influence which prejudice and passion have over the opinions of mankind will, frequently pity the aberrations of intellect, where others see only objects of unmingled censure. When we reflect that wherever there is any room for the play of passion, the majority of the world judge rather from prejudice than from reason—that innovators in religion and politicks, as well as the enemies of both, in endeavoring to overturn established institutions, have always aimed rather at the vices of their advocates than the falsity of their opinions—that false principles of honor have dissolved every social obligation, and been made to supply the place of every virtue—that religion, whose very essence consists in the cultivation of benevolent feelings, has called to her aid instruments of torture, and that the strongest obligations, and the tenderest ties have been sacrificed to the cruel commands of an imaginary Deity, and hecatombs of human victims offered upon his altars—when we reflect upon the melancholy instances of this nature which fill the pages of history, we will frequently be led to soften the asperity of our censure, and to ascribe to education, prejudice or fashion, what others attribute to moral obliquity. Nor will we be surprised if persons of warm sensibilities, whose education has been directed to objects of superficial ornament rather than of practical utility, or whose imagination or religious feelings have been highly cultivated, without being corrected by intercourse with the world, or habits of active industry, should have their judgment so distorted as to unfit them for the duties of life.

Not only is the judgment liable to be perverted by the imagination, but the passions frequently mislead it. Such is the influence of passion in stifling the voice of reason, that the common law very properly extenuates acts of violence committed under the excitement of sudden insult, or personal injury. And so completely is the voice of reason supposed to be hushed under the presure of certain injuries, that public opinion would justify almost any act of violence, although public expediency might require the punishment of the crime.

The case of an individual is truly unfortunate whom the passions and the imagination combine to mislead. Such are those persons in whom time and reflection increase instead of diminishing the sense of injury—in whom a suspicious and ever active imagination, by continually introducing a thousand imaginary grievances, excites a spirit of unrelenting hostility towards the most valuable friends. Such was the character of the celebrated Rousseau, a writer of the liveliest imagination, the finest talents, and the warmest heart, but who by his unreasonable suspicions, alienated the affection of his friends, and rendered his own life miserable.

The man must be strangely ignorant of the principles of human nature, or destitute of the finest principles of humanity, who can look upon the conduct of these persons with any other feelings than those of sympathy and kindness; or who by resenting their conduct, could add to the unhappiness of their condition. How far considerations of this kind should influence the principles of the law in criminal cases, is a very intricate and difficult question. Yet many reasons might be assigned why the same humane principles which regulate our intercourse with the world, should be applied to the administration of the criminal law.

Individuals are frequently hurried away by an uncontrollable impulse—a morbid animal feeling, to commit acts of violence upon themselves or others. Such persons in their rational intervals are frequently aware of this propensity to mischief, and request to be confined to prevent its consequences. These individuals frequently conduct their designs with the greatest art and secrecy, and after the commission of an act of violence, generally take precautions to escape detection, or avoid punishment. The animal feelings in insanity are always in excess, and cowardice therefore, except when the nature of the delusion leasd to contempt of danger, is one of its characteristicks. The mono-maniac is fully sensible that the opinion of the world is against him, and his disease necessarily implies a knowledge of the consequences of crime. Nothing therefore could be more erroneous than the mode of reasoning very general, among even intelligent members of society, that because an individual conducts himself in the management of a criminal enterprise with caution and secrecy, he must necessarily be a moral agent, and amenable to the laws of his country.

In connection with this subject, we will merely offer a suggestion in relation to an opinion generally prevalent, and received as a settled principle in criminal jurisprudence; which is, that monomania can be urged in extenuation of those crimes only, which are connected with the subject of the delusion. We do not pretend to lay down a general rule, or say how far the extenuation should be carried, but simply to remark that a monomaniac, however well he may occasionally converse and act, seldom has his judgment, imagination, passions, and affections sufficiently balanced, to constitute him a perfect moral agent; and the legal principle has long been settled, that no considerations of expediency should ever make an individual suffer for crimes, of which he is not *or cannot* be guilty—a principle as honorable to the good sense as it is to the good feelings of the community in which it originated. It never can be good policy to inflict a punishment which would be revolting to the sentiments of enlightened humanity.

We shall say nothing of the obliquities of the moral sentiment. It is difficult to distinguish them from the effects of bad education and vicious habits, (indeed they often arise from these) and to these the policy of our laws extends no indulgence. The subject has never received the attention it merits.

We shall conclude these desultory, and too protracted remarks, by an additional extract from the same Review.

The Editor of this Journal was very much amused, one day, while visiting the Lunatic Asylum, in Florence, (where it is the custom to send all insane people, whatever their station in life,) by the conversations of a Priest and an Advocate, who were monomaniacs of such a harmless description that the keeper permitted them to accompany the Editor round the whole of the wards and cells of that great and wretched asylum. These two inmates of this gloomy retreat were men of considerable talents and learning. They described in most affecting terms, the various maniacs who paced the wards in musing melancholy or muttering soliloquies, as well as those who clashed their chains in solitary confinement. Not a word escaped either of them, in the slightest degree indicative of a disordered mind, till they came to a man who fancied himself to be Jesus Christ. The Barrister made a full stop, and seized the writer by the arm. "Thahk my stars," said he, glancing a look of ineffable contempt on the Priest, "I am free from those superstitious fears and visionary dreams by which the vulgar are kept in thraldom by designing knaves or ignorant enthusiasts! I worship the sun, the moon, the stars, the earth—in short, I worship Nature, whatever form she may assume in the animal, vegetable, and mineral world around me, as well as in those orbs which shine resplendent in the heavens. I acknowledge no god but Nature."—At this moment, the Priest seized the other arm of the writer, and drew him forcibly aside. "You now see, Sir," said he, "that the unhappy and lost wretch who deals out this impious and atheistical creed is as complete a maniac as any of the numerous unfortunate beings whom we have been contemplating! He is otherwise harmless; but his words are pestilential, when he touches on the subject of divine revelation. I am, Sir, the only individual in this vast asylum, who is in his perfect senses. I am cruelly and unjustly confined here, and, as I see you are a physician, I hope you will exert your influence in rescuing me from the company of maniacs." The writer promised this exertion in the Priest's favour, but soon found that he, too, had his delusion. It turned out that the Barrister was not so insane upon religious topics as on some others. His insanity was first discovered by a pathetic love-letter to a beautiful young English lady at Florence! It is notorious that the insane are perfectly conscious of the madness of their comrades, while they are totally blind to their own. But is not this the case with every eccentricity—even obliquity of intellect—every failing of human nature? F.

